# Deafness mutation mining using regular expression based pattern matching

**DOI:** 10.1186/1472-6947-7-32

**Published:** 2007-10-25

**Authors:** Christopher M Frenz

**Affiliations:** 1 Department of Computer Engineering Technology, New York City College of Technology (CUNY), 300 Jay St, Brooklyn, NY 11201, USA

## Abstract

**Background:**

While keyword based queries of databases such as Pubmed are frequently of great utility, the ability to use regular expressions in place of a keyword can often improve the results output by such databases. Regular expressions can allow for the identification of element types that cannot be readily specified by a single keyword and can allow for different words with similar character sequences to be distinguished.

**Results:**

A Perl based utility was developed to allow the use of regular expressions in Pubmed searches, thereby improving the accuracy of the searches.

**Conclusion:**

This utility was then utilized to create a comprehensive listing of all DFN deafness mutations discussed in Pubmed records containing the keywords "human ear".

## Background

Biological research has yielded a vast amount of research data, which can often provide novel insights when the data can be viewed in an aggregated fashion, and thus recent studies have employed computational methods of information extraction from the biomedical literature. These studies have dealt with a wide range of information extractions, including the names of genes and proteins[1], intermolecular relationships [2], and molecular biological descriptors [3].

Pubmed currently catalogs citation and abstract information for over 4,400 biomedical research journals and houses a citation database of over 12.8 million citations[4]. With any database of this size the return of relevant query results is often a difficult task, given the large number of potential matches there likely are for any single query term. These difficulties are compounded even further, given that Pubmed records are all natural language records and searches cannot readily be conducted using a predefined set of terms, as is the case for many relational databases. Thus Pubmed employs a word-matching algorithm, which seeks to match query words to the contents of citation records, and will return all records containing that word in their order of publication starting with the most recent.

For certain types of queries, such as mutations, basic word matching is an ineffective search strategy, since an effective query cannot be specified as a single word, but rather is better expressed as a textual pattern, such as [Residue] [Position] [MutantResidue] [5]. The use of textual pattern matching, however, has a wide array of uses that extend beyond the location of mutations within Pubmed records, and include the ability to distinguish between articles which discuss pKa values as opposed to articles that discuss Protein Kinase A (PKA), which would both be yielded by a Pubmed search for the "pKa" word. These above examples, illustrate the two major applications that text patterns offer to Pubmed searching; 1) the identification of elements that cannot be specified by a single word and 2) distinguishing between two different words that are comprised of a similar sequence of characters [6]. Textual patterns are commonly matched via the use of regular expressions and studies that involve the extraction of biochemical mutation data from biomedical literature have demonstrated a high degree of success [5,7]. This study seeks to develop a Perl based utility Perl Regular Expressions for Pubmed (PREP.pl, See Additional File 1, which allows the searching of Pubmed citation records for the presence of textual patterns and for the placement of match containing records into an HTML formatted output file. This Perl based utility will then be utilized to construct a comprehensive listing of DFN mutations discussed in Pubmed records containing the "human ear" keywords.

## Implementation

### The PREP utility

The script interacts with Pubmed via NCBI's E-Utilities interface [4] and the LWP module handles all HTTP based communication. The script begins by using the ESearch method to query Pubmed for all records containing a user defined search term, such as "lysozyme" or "HIV". Pubmed ID numbers of all matching records are temporarily stored on the Pubmed server and can be accessed using the EFetch method and an assigned Web environment variable and query key, which is returned by the ESearch method. Records returned by the EFetch method are requested in XML format, since the well-defined hierarchical structures of XML documents greatly simplifies parsing tasks [6]. This script makes use of the XML::LibXML Perl module for XML parsing, and from each Pubmed record the title of the article, the journal information, the abstract, and the Pubmed ID of the record, are extracted, based on their corresponding XML tag names.

**Figure 1:**
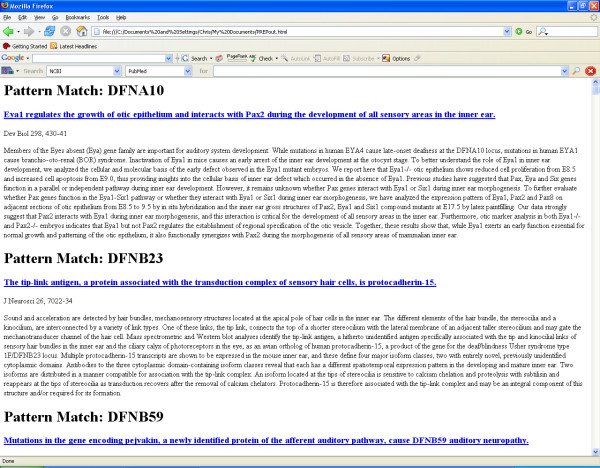
A screen shot of the PREP program output for a search for DFN deafness.

A user specified regular expression is then used to search the abstract and title fields of each record and look for a textual pattern match. Only the title and abstract fields are searched, since these are the fields in which pattern matches are most likely to be found, and the elimination of other fields reduces the potential for false positives. If a match occurs the journal information, the abstract, and title are output to an HTML file (Figure 1).

The title is output in the format of a hyperlink to the Pubmed record that corresponds to that article, to allow for easy retrieval of any additional information pertaining to the article that the output file does not provide or in certain cases easy retrieval of the entire article. The generation of an HTML output allows for the results to be easily shared among users working on disparate computing platforms. Records that contain no matches to the text pattern of interest are not written to an output file. On an AMD Athlon 2000+ the PREP script can process an average of 500 abstracts per minute.

PREP can be run from the command line of any Linux or Unix machine that has the XML::Lib::XML Perl module installed. The regular expression used within the script is modified by changing the value of the $regex variable within the script, as indicated by the code documentation. Command line script execution can be initiated using the standard Perl command line syntax of "perl Prep.pl Keywords". The utility was chosen to be implemented in a command line fashion since this makes the utility suitable for easy inclusion in more comprehensive data mining scripts where the search functionality of PREP may provide useful.

### Validation of utility

As a test of the specificity obtainable by the PREP script, all Pubmed records that resulted from a search for the word "lysozyme" were checked for pattern matches to the Protein Kinase A abbreviation "PKA" by using the regular expression PKA within the PREP script. At the time the test was conducted there were 19,964 records returned, and the PREP script indicated that only 3 records contained the textual pattern "PKA". These findings were manually confirmed by going through all records returned by the lysozyme search. The textual pattern "pKa", however, is actually fairly common throughout the lysozyme record set, and the PREP script successfully eliminated these "pKa" containing records from the search results, whereas the standard Pubmed keyword search is unable to accomplish this. Thus, this test is indicative that with a well-formed regular expression a high degree of specificity and search refinement can be achieved between different words with like character compositions. While no false positives were noted during the manual confirmation of these search results, the potential source of false negatives for this search would be abstracts that discussed Protein Kinase A without mentioning the abbreviation PKA.

The ability of the PREP script to identify elements that cannot be specified as a single word was tested by searching for mutations in the records returned by a search for "hen egg white lysozyme" using the regular expression:

[ARNDCEQGHILKMFPSTWYV]\d+[ARNDCEQGHILKMFPSTWYV]|

[A-Z][a-z][a-z]\d+[A-Z][a-z][a-z]

This expression allows for the identification of mutations written out in both the single letter amino acid notation as well as the three-letter notation. The "hen egg white lysozyme" search of Pubmed yielded 1146 records of which PREP identified 62 as matching the above regular expression pattern, and were manually confirmed. Of these 62 matches, 36 (58%) records contained actual mutations while the remaining 42% contained false positives, such as the abbreviation for T4 Lysozyme (T4L). In order to lessen the percentage of false positives, the false positives were examined and it became apparent that many of the same false positives occurred in repeated records. Thus a simple filter was created by defining a second regular expression, which explicitly matched the false positives, and prevented them from being recorded in the program output, thereby eliminating these repeating false positives. In this manner, the total number of PREP matches was reduced to 47, raising the percentage of valid positives up to 77% and reducing the number of false negatives to 23%. This is indicative that the PREP script can be an effective tool in reducing the search space necessary for manual processing by taking the 1146 initial records and narrowing down the list of possible records to 47, or 4% of the original search space. It should be further noted that the PREP script did not miss any records that contained matching patterns within the data set, and that the DFN prefix associated with deafness mutations is less likely to turn up false positives than the more generalized pattern associated with biochemical mutation data. This validation exercise, though, does demonstrate the utility of an application specific filter as a means of reducing false positives where warranted.

## Results

The textual pattern DFN [A-Z]\d+ was defined, where [A-Z] could be any letter between A and Z and \d+ could be a combination of one or more numeric digits and this pattern used to search through records returned by a PubMed search for the keywords "human ear". The search yielded 61,371 Pubmed records and out of those 117 contained a pattern match. All of the pattern matches corresponded to valid DFN deafness mutations and no false positives were returned. The DFN mutation found in the 117 matching records are summarized in Table 1. In cases where multiple records discussed a mutation, a representative record is listed in the source field, rather than every record, to limit table length.

**Table 1:** Mutations located in the PREP program results

**Mutation Name**	**Mutation Effect**	**Representative Source**
DFNA1	Diaphonous gene mutation associated with autosomal dominant non-syndromic hearing loss	9
DFNA10	Mutation in EYA4 causes late onset deafness	10
DFNA11	MYO7A mutation that results in progressive loss of mechanotransduction	11
DFNA12	TECTA mutation resulting in hearing impairment	12
DFNA13	Mutation leading to cochlear conductive loss	13
DFNA14	wolframin mutation cauisng non-syndromic dominant low frequency hearing loss	14
DFNA15	Mutation in POU4F3 that leads to autosomal dominant non-syndromic hearing loss	15
DFNA17	Mutation in myosin heavy chain IX linked to hearing impairment	16
DFNA2	KCNQ4 potassium channel mutation leading to progressive hearing loss	17
DFNA20	ACTG1 mutation causing autosomal dominant heairng loss	18
DFNA24	Caspase 3 mutation associated with autosomal dominant non-syndromic hearing loss	19
DFNA26	ACTG1 mutation causing autosomal dominant heairng loss	18
DFNA36	Mutation in transmembrane cochlear expressed gene 1 causing progressive deafness	20
DFNA38	wolframin mutation cauisng non-syndromic dominant low frequency hearing loss	14
DFNA39	Hearing loss associated with Dentinogenesis imperfecta	21
DFNA4	MYH14 mutation leading to autosomal dominant hearing loss	23
DFNA48	MYO1A mutation resulting in autosomal dominant hearing loss	22
DFNA5	Mutation causing autosomal dominant hearing impairment	24
DFNA6	wolframin mutation cauisng non-syndromic dominant low frequency hearing loss	14
DFNA8	TECTA mutation resulting in hearing impairment	12
DFNA9	Coagulation factor C homology gene mutations causing sensioneural hearing loss	25
DFNB1	Connexin 26 mutation leading to non-syndromic hearing loss	26
DFNB11	Mutation in transmembrane cochlear expressed gene 1 causing congenital deafness	20
DFNB12	Cadherin 23 mutation causing prelingual hearing loss	14
DFNB13	Mutations causing autosomal recessive non-syndromic deafness	27
DFNB14	Hearing loss associated with split hand/split foot malformation	28
DFNB16	Stereocilan mutation leading to autsomal recessive non-syndromic deafness	29
DFNB17	FAM3C mutation causing autosomal recessive non-syndromic hearing loss	30
DFNB18	Deafness associated with Usher syndrome 1C	31
DFNB2	Deafness associated with mutations in myosin VIIA gene	32
DFNB22	Otoancorin mutation resulting in autosmal recessive deafness	33
DFNB23	Usher Syndrome 1F related deafness	34
DFNB25	Chromosome 5 mutation that effects sensory mechanotransduction	35
DFNB28	TRIOBP mutation resulting in recessive prelingual sensioneural hearing loss	36
DFNB29	CLDN14 mutations resulting in autosomal recessive non-syndromic deafness	37
DFNB3	Myo15a related non-syndromic deafness	38
DFNB30	Mutation in myosin IIIA resulting in progressive hearing loss	39
DFNB31	Whirlin mutation resulting in hearing loss	18
DFNB4	Mutation in PDS gene causing congenital deafness	40
DFNB59	Autosomal recessive auditory neuropathy	41
DFNB6	Mutation causing autosomal recessive deafness	42
DFNB67	Mutation in THMS causing recessive non-syndromic hearing loss	43
DFNB7	Mutation in transmembrane cochlear expressed gene 1 causing congenital deafness	20
DFNB8	TMPRSS3 mutation associated with non-syndromic autosommal recessive hearing loss	44
DFNB9	Mutation in otoferlin causing prelingual hearing loss	45

## Discussion & conclusion

The PREP script was able to process 61,371 Pubmed records displaying the keywords "human ear" and narrow the relevant search space down to 117 articles that contain different DFN deafness mutations. This is slightly less than 0.2% of the original search space, demonstrating the utility of pattern matching in aiding researchers in obtaining relevant information from the biomedical literature. Furthermore, the lack of false positives among the returned results demonstrates that the accuracy and utility of this approach can be further enhanced when the defined pattern possesses a high degree of specificity. The PREP approach to literature searching would therefore allow researchers to uncover a diversity of information pertaining to deafness mutations in a single search, whereas uncovering the same 45 DFN deafness mutations (Table 1) by standard keyword searches would take considerably more time and effort. However, when utilizing such an approach to literature searching, in addition to false positives, it is important to carefully consider the keywords presented to Pubmed. For example, this "human ear" keyword search failed to uncover the DFNB35 mutation [8] since it does not appear in an abstract that contains the words "human" and "ear". Potential sources of false negatives among search results include papers that do not utilize the DFN based nomenclature to discuss the mutation or articles that mention the abbreviation in the text, but not the abstract. Based on the validation tests, however, the false negative rate is expected to be low. Even with these limitations, however, the textual pattern based search methodology presented here can be of great value to researchers in the otolaryngological sciences as well as in other biomedical disciplines, since regular expressions can also be created to match other biological patterns, such as DNA or protein sequences, ions, enzyme names, and numerous other possibilities.

## Availability & requirements

Project Name: PREP: Perl Regular Expressions for PubMed

Project Home Page: 

Operating Systems: Linux/Unix

Programming Language: Perl

Other Requirements: XML::LibXML Perl Module

License: Perl Artistic License

## Competing interests

The author(s) declare that they have no competing interests.

## Authors' contributions

CMF is responsible for the study and manuscript in their entirety.

## Pre-publication history

The pre-publication history for this paper can be accessed here:



## Supplementary Material

Additional file 1 The PREP.pl Perl script. Click here for file
